# Clinical impact of primary and secondary *KIT* mutations on the efficacy of molecular-targeted therapies in gastrointestinal stromal tumors

**DOI:** 10.1007/s10120-025-01639-1

**Published:** 2025-07-23

**Authors:** Kota Kawabata, Tsuyoshi Takahashi, Toshirou Nishida, Yukinori Kurokawa, Kazuyoshi Yamamoto, Takuro Saito, Kota Momose, Kotaro Yamashita, Koji Tanaka, Tomoki Makino, Ryohei Kawabata, Atsushi Takeno, Kiyokazu Nakajima, Seiichi Hirota, Hidetoshi Eguchi, Yuichiro Doki

**Affiliations:** 1 Department of Gastroenterological Surgery, Osaka University Graduate School of Medicine, 2-2, Yamadaoka, Suita, Osaka, 565-0871 Japan; 2 Department of Surgery, Japan Community Health Care Organization Osaka Hospital, 4-2-78, Fukushima, Fukushima-ku, Osaka, Japan; 3 Department of Surgery, Sakai City Medical Center, Ebarajicho, Nishi-ku, Sakai, Osaka, Japan; 4 Department of Surgery, NHO Osaka National Hospital, 2-1-14, Hoenzaka, Chuo-ku, Osaka, Japan; 5 Department of Surgical Pathology, Hyogo Medical University School of Medicine, Nishinomiya, Japan

**Keywords:** Gastrointestinal stromal tumors, Gene mutations, Genetic profiling, Molecular-targeted therapy, Tyrosine kinase inhibitors

## Abstract

**Background:**

Gastrointestinal stromal tumors (GISTs) are commonly driven by primary mutations in *KIT* or *PDGFRA*. Imatinib is the first-line therapy for GISTs. However, secondary mutations frequently emerge during imatinib treatment, contributing to resistance and influencing the efficacy of subsequent tyrosine kinase inhibitors, such as sunitinib and regorafenib. This study aimed to investigate the clinical relevance of both primary and secondary *KIT* mutations in treating and prognosing unresectable or recurrent GISTs.

**Methods:**

Ninety patients with unresectable or recurrent GISTs treated at our institution between 2000 and 2017 were retrospectively analyzed. Genetic testing was performed before the initial drug administration to guide first-line imatinib therapy based on the primary mutation profile. In 64 imatinib-resistant patients, additional genetic testing was conducted on tissues obtained from resistant lesions. Treatment response and prognosis were compared across mutational profiles.

**Results:**

The most common primary mutation was *KIT* exon 11 (76.7%), followed by exon 9 (12.2%). Patients with exon 9 mutations showed superior progression-free survival with sunitinib than those with exon 11 mutations. Among patients with exon 11 primary mutations, secondary mutations were identified in 79.2%, predominantly in *KIT* exon 13/14 (47.9%) or 17/18 (31.3%). Sunitinib was more effective in patients with secondary exon 13/14 mutations, whereas regorafenib was significantly more effective in those with exon 17/18 mutations.

**Conclusions:**

Secondary *KIT* mutations play a crucial role in imatinib resistance and the efficacy of second- and third-line therapies. Genetic profiling at the initial diagnosis and at the time of resistance may provide more personalized and effective treatment strategies.

**Supplementary Information:**

The online version contains supplementary material available at 10.1007/s10120-025-01639-1.

## Introduction

Gastrointestinal stromal tumors (GISTs), the most prevalent stromal tumors in the gastrointestinal tract, are a type of sarcoma that originates from the precursor interstitial cells of Cajal [1]. GISTs are primarily caused by the constitutive activation of the receptor tyrosine kinases *KIT* and platelet-derived growth factor receptor A (*PDGFRA*), which are mainly associated with gain-of-function mutations in the *KIT* and *PDGFRA* genes [2, 3]. The primary treatment for unresectable or recurrent GISTs is with tyrosine kinase inhibitors (TKIs), such as imatinib, which target these genetic mutations [4]. Imatinib is a selective *KIT* inhibitor with proven clinical efficacy that has been established as the first-line treatment for unresectable or recurrent GISTs [5]. The median progression-free survival (PFS) has been reported to exceed 2 years, but most GISTs become resistant to imatinib [6]. In cases of imatinib resistance, other TKIs, such as sunitinib, regorafenib, and ripretinib, are used, and recently, pimitespib, which has a different mechanism of action, has been developed [7–10]. However, the prognosis of imatinib-resistant GIST remains poor, highlighting the necessity to identify established indicators for evaluating treatment efficacy and prognosis.

Several resistance mechanisms to molecular-targeted agents, including TKIs, have been proposed, such as target resistance due to mutations or genomic as well as protein amplification, biological resistance, and functional resistance, such as pharmacokinetic changes [11]. Previous studies have reported that secondary mutations in *KIT* or *PDGFRA* are the main cause of imatinib resistance in patients with unresectable or recurrent GISTs [12]. When secondary mutations occur in these genes, it has been reported that the type of mutation affects the response to other TKIs, such as sunitinib and regorafenib, mainly based on in vitro data [13]. Recently, several studies have suggested that the location of secondary *KIT* mutations may affect the efficacy of TKI therapies after imatinib treatment [14, 15]. Based on these findings, a clinical trial aiming to develop personalized therapies targeting specific secondary mutations is also being conducted [16]. However, in patients with unresectable or recurrent GISTs, where a multi-line TKI treatment strategy from first-line to fourth-line therapy has been established, it remains unclear to what extent such treatment strategies contribute to overall prognosis. Furthermore, given the rarity of GIST and the limited number of patients who acquire imatinib resistance, there is a critical need to gather real-world clinical data to elucidate both the therapeutic efficacy of each agent based on mutational status and the validity of sequential treatment strategies in this context. At our institution, when a patient with unresectable or recurrent GISTs acquires imatinib resistance, surgical resection is considered a treatment option if the resistant lesion is partial. Furthermore, our institution has been conducting genetic testing of imatinib-resistant lesions obtained through resection and has accumulated long-term data.

Although secondary mutations in GIST-related genes are associated with treatment resistance in patients with unresectable or recurrent GISTs, the incidence of secondary gene mutations, response to subsequent drug treatment, and prognosis remain unclear in the real world. Therefore, we aimed to clarify the types and frequencies of GIST-related gene mutations in unresectable or recurrent GISTs and determine their clinical value by focusing on the relationship between gene mutations, therapeutic effects of each drug, and prognostic implications.

## Materials and methods

### Participants and methods

This retrospective study analyzed the data of patients diagnosed with unresectable or recurrent GISTs at the Osaka University Hospital between January 2000 and December 2017. Imatinib was initially administered to the patients. Subsequently, if the patient became resistant to imatinib, they were treated with other TKIs (sunitinib as second-line treatment and regorafenib as third-line treatment). Cases that failed or were intolerant to these TKIs were treated with pimitespib, a heat shock protein 90 (HSP90) inhibitor. If the number of imatinib-resistant lesions was limited to one or two, patients were given the option of surgical resection followed by imatinib until they were diagnosed with systemic resistance. The collected data included patient characteristics, primary and metastatic site details, genetic information, and prognostic factors. The data cutoff was set for February 2025. This retrospective study was approved by the Human Ethics Review Committee of the Osaka University Graduate School of Medicine (Approval ID: 22,316). All patients provided written informed consent for the use of their clinical data before treatment. All procedures were performed in accordance with the ethical standards of the responsible committee on human experimentation (institutional and national) and the Helsinki Declaration of 1964 and later versions.

### Genetic testing

All patients were tested for GIST-related genes using tissue samples from radical resection of the primary tumor as the initial treatment, or from resection or biopsy of unresectable or recurrent metastatic lesions. Furthermore, genetic testing was performed on tissue samples from resected or biopsied imatinib-resistant lesions. Genetic testing of GISTs was performed using sequence analysis based on reverse transcription-polymerase chain reaction (RT-PCR), direct Sanger sequencing, or gene panel testing using next-generation sequencing (NGS).

### Sequence analysis based on RT-PCR

Fresh samples were snap-frozen in liquid nitrogen at the time of surgical resection or biopsy and were stored at −80 °C until RNA extraction. Total RNA was extracted using an RNeasy Mini Kit (Qiagen, Valencia, CA, USA). cDNA was synthesized using reverse transcriptase (Superscript II; Gibco BRL, Grand Island, NY, USA). *KIT* or *PDGFRA* cDNA was amplified using RT-PCR and sequenced, as previously described [17]. The samples, including the known sites of *KIT* (exons 8, 9, 11, 13, 14, 17, and 18) and *PDGFRA* (exons 12, 14, and 18) mutations, were tested.

### Genetic testing using direct Sanger sequencing

*KIT* mutations were analyzed using direct Sanger sequencing of imatinib-resistant lesions in the patients already known to have a primary *KIT* mutation. The fresh samples that were snap-frozen in liquid nitrogen, or formalin-fixed, paraffin-embedded, thin-sectioned samples retrieved from tumor specimens were delivered and sequence analysis was performed by LSI Medience (#45,416, Tokyo, Japan, https://www.medience.co.jp/). These samples, including the known sites of *KIT* (exons 8, 9, 11, 13, 14, 17, and 18) mutations, were tested.

### Gene panel testing using NGS

Comprehensive genomic analyses were performed using FoundationOne® CDx (F1CDx; Foundation Medicine, Inc.). Formalin-fixed, paraffin-embedded, thin-sectioned samples retrieved from tumor specimens were delivered to Foundation Medicine, Inc. F1CDx applied NGS to 324 genes known to be drivers of solid tumors with high accuracy. Sequencing was performed using an Illumina HiSeq® 4000 (Illumina) to identify base substitutions, insertions and deletions, copy number alterations, and rearrangements. F1CDx covered the entire exonic region of *KIT* and *PDGFRA* for mutation determination.

### Statistical analyses

Data were analyzed using the JMP17 software (SAS Institute Inc., Cary, NC, USA). Continuous data are presented as medians and ranges. Group differences were analyzed using the chi-square and Mann–Whitney U tests. Patient survival was analyzed using Kaplan–Meier curves and log-rank tests of differences. PFS was defined as the time from treatment initiation to either disease progression or death from any cause, whichever occurred first. Overall survival (OS) was calculated from the date of diagnosis of unresectable or recurrent GISTs, the initiation date of each drug, or the date of the first documented imatinib resistance to the last date of confirmed survival. Patients who were alive on the date of the last follow-up were censored. Statistical significance was set at p < 0.05.

## Results

### Patient characteristics

**Fig. 1:**
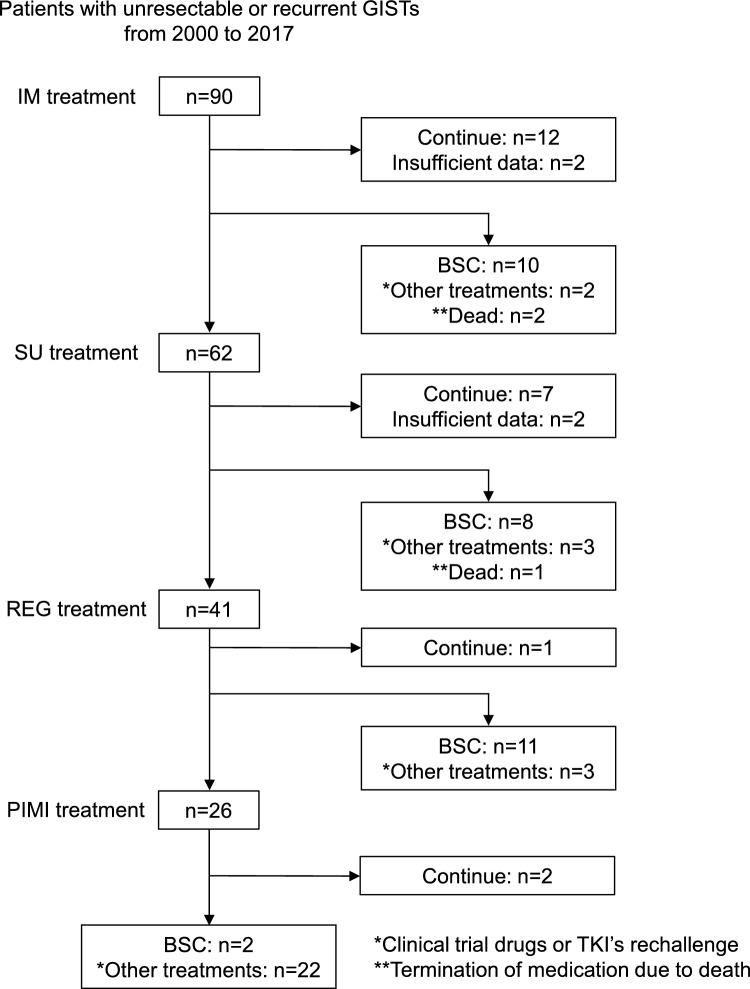
Patient flowchart. GISTs, gastrointestinal stromal tumors; IM, imatinib; SU, sunitinib; REG, regorafenib; PIMI, pimitespib; BSC, best supportive care; TKI, tyrosine kinase inhibitor

Between 2000 and 2017, 90 patients diagnosed with unresectable or recurrent GISTs at the Osaka University Hospital underwent GIST-related genetic testing using tissue samples collected before imatinib treatment (Fig. 1). Table 1 shows the patient characteristics. The median age of the patients was 59 (range, 17–82) years, 57.8% were male, and 84.4% had an Eastern Cooperative Oncology Group performance status of 0. The most common primary site of GISTs was the small intestine (n = 48, 53.3%). Most metastases were heterochronous (n = 69, 76.7%). Neoadjuvant chemotherapy and adjuvant chemotherapy with imatinib were administered in 2.9% and 37.7% of patients, respectively. The liver (n = 41, 45.6%), peritoneum (n = 34, 37.8%), and both (n = 13, 14.4%) were the common sites of metastasis. Imatinib-resistant lesions were resected in 61 patients (67.8%).

**Table 1:** Patient characteristics

Age^*^, years	n = 90	
Median (range)	59	(17–82)
Sex, *n* (%)		
Male	52	(57.8)
Female	38	(42.2)
ECOG PS*		
0	76	(84.4)
1	14	(15.6)
Location of primary lesion, *n* (%)		
Esophagus	1	(1.1)
Stomach	33	(36.7)
Small intestine	48	(53.3)
Rectum	7	(7.8)
Others	1	(1.1)
Primary gene mutation, *n* (%)		
* KIT* exon 11	69	(76.7)
* KIT* exon 9	11	(12.2)
* PDGFRA* exon 18	4	(4.4)
Wild type	6	(6.7)
Genetic testing method^**^, *n* (%)		
RT-PCR	82	(91.1)
Gene panel testing	8	(8.9)
Format of metastasis, *n* (%)		
Heterochronous metastasis	69	(76.7)
Concurrent metastasis	21	(23.3)
Neoadjuvant chemotherapy with IM		
Presence	2	(2.9)
Absence	67	(97.1)
Adjuvant chemotherapy with IM		
Presence	26	(37.7)
Absence	43	(62.3)
Location of metastatic lesion, *n* (%)		
Liver	41	(45.6)
Peritoneum	34	(37.8)
Liver and peritoneum	13	(14.4)
Others	2	(2.2)
Number of TKIs		
1	28	(31.1)
2	21	(23.3)
3 or high	41	(45.6)
Resection of IM-resistant lesions, *n* (%)		
Yes	61	(67.8)
No	29	(32.2)

*Age or ECOG PS at diagnosis of unresectable or first recurrence

**The method used prior to the initiation of IM treatment

ECOG PS: Eastern Cooperative Oncology Group performance status, RT-PCR: reverse transcription-polymerase chain reaction, TKIs: tyrosine kinase inhibitors, IM: imatinib

**Fig. 2:**
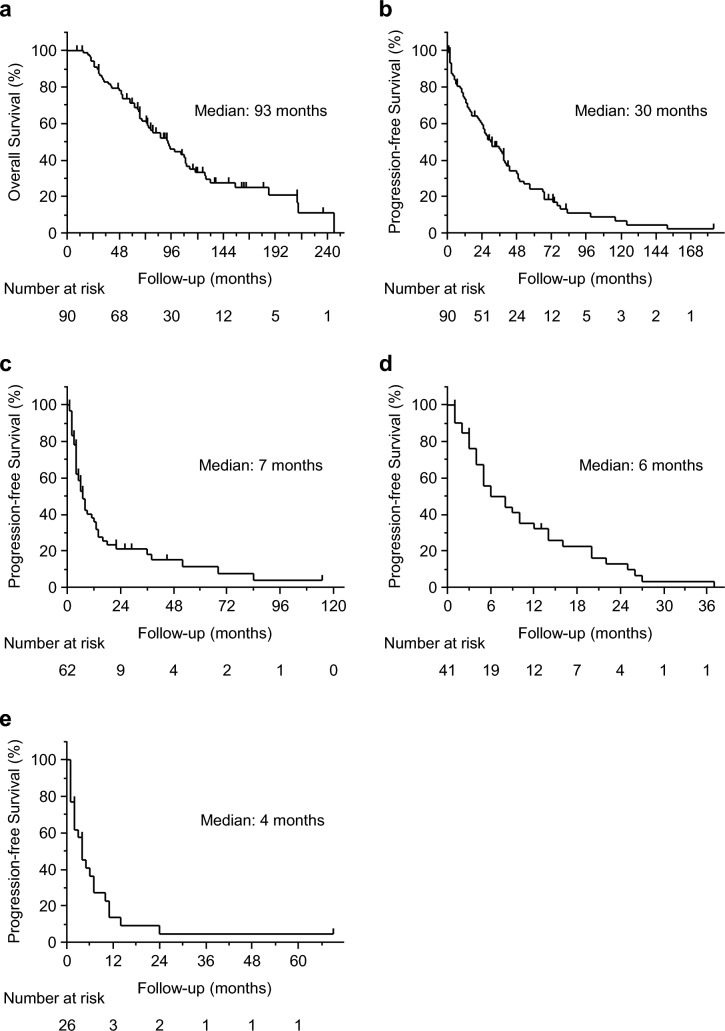
Kaplan–Meier curves for survival. **a** Overall survival after diagnosis of unresectable or recurrent gastrointestinal stromal tumors (n = 81). Progression-free survival for **b** imatinib (n = 81), **c** sunitinib (n = 52), **d** regorafenib (n = 35), and **e** pimitespib (n = 19)

Of the 90 patients who were initially treated with imatinib after the diagnosis of unresectable or recurrent GISTs, 12 continued imatinib treatment (Fig. 1). Following imatinib therapy, 10 patients transitioned to the best supportive care, while the remaining 62 proceeded to second-line treatment with sunitinib. Among the 62 patients, seven continued sunitinib therapy. Upon discontinuation of sunitinib, eight patients transitioned to best supportive care, while 41 advanced to third-line treatment with regorafenib. Among the 41 patients, one remained on regorafenib therapy. Following the discontinuation of regorafenib, 11 patients transitioned to the best supportive care, while 19 proceeded to the fourth-line treatment with pimitespib. The median OS after the diagnosis of unresectable or recurrent GISTs was 93 (range, 9–246) months (Fig. 2a). There were 54 deaths due to exacerbation of the primary disease and six deaths from other causes. The median PFS for imatinib, sunitinib, regorafenib, and pimitespib were 30 (range, 1–184), 7 (range, 1–115), 6 (range, 1–37), and 4 (range, 1–69) months, respectively (Fig. 2b–2e).

### Clinical characteristics by primary gene mutation

**Fig. 3:**
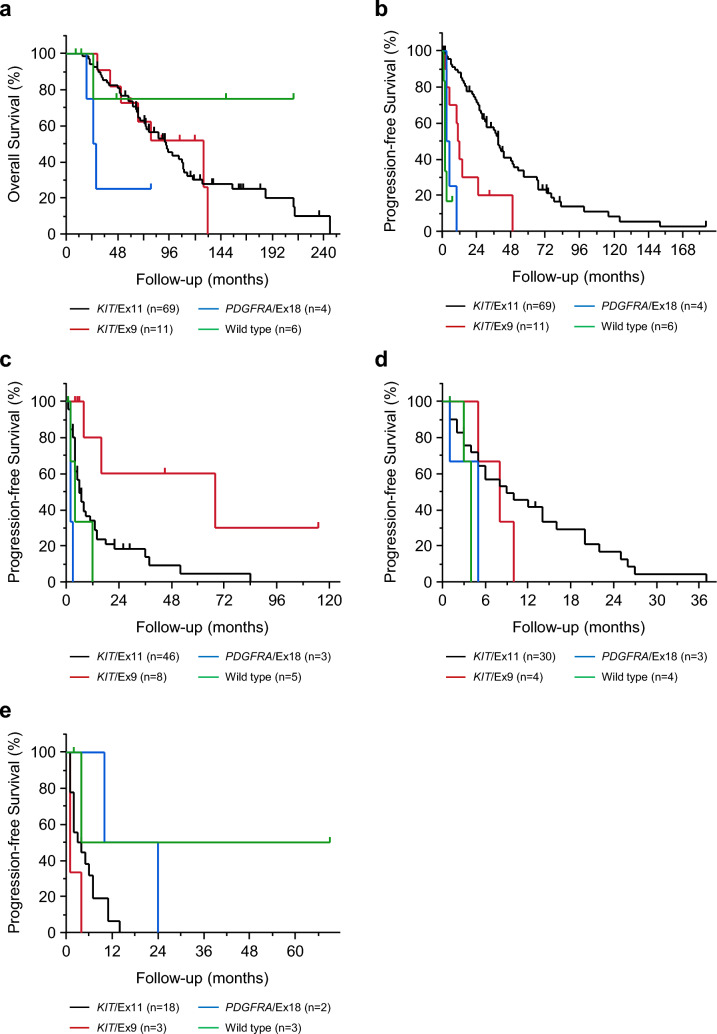
Kaplan–Meier curves for survival by primary gene mutation. **a** Overall survival after diagnosis of unresectable or recurrent gastrointestinal stromal tumors. Progression-free survival for **b** imatinib, **c** sunitinib, **d** regorafenib, and **e** pimitespib. Survival rates were compared using the log-rank test

Table 2 shows the characteristics of the 90 patients who were divided according to primary mutations in GIST-related genes. The primary mutations were in exon 11 (*KIT*/Ex11 group; n = 69, 76.7%) and exon 9 (*KIT*/Ex9 group; n = 11, 12.2%) of the *KIT* gene, exon 18 of the *PDGFRA* gene (*PDGFRA*/Ex18 group; n = 4, 4.4%), and *KIT*/*PDGFRA* wild-type (Wild type group; n = 6, 6.7%). The median age of the Wild type group was 35 (range, 17–54) years, which was lower than that of the other groups. The proportion of the small intestine as the primary site was higher in the *KIT*/Ex9 group (n = 10, 90.9%) than in the other groups. No other characteristics showed significant differences between the groups. There were no clear differences between the four groups in the OS after the diagnosis of unresectable or recurrent GISTs (Fig. 3a). Similarly, no clear differences in OS for each drug administered after second-line treatment were observed among the groups (Supplementary Fig. 1). The median PFS with imatinib was 39 (range, 1–184) months in the *KIT*/Ex11 group, which was significantly longer than those in the other groups (all p < 0.001; Fig. 3b). The median PFS with sunitinib was 68 (range, 6–115) months in the *KIT*/Ex9 group, which was significantly longer than those in the *KIT*/Ex11 group (p = 0.007; Fig. 3c). Although there was no significant difference, the PFS of regorafenib in the *KIT*/Ex11 group tended to be longer than that in the other groups (Fig. 3d). There was no significant difference in PFS with pimitespib among the four groups (Fig. 3e).

**Table 2:** Comparison of patient characteristics with known primary gene mutation

Age^*^, years	*KIT*/Ex11 n = 69		*KIT*/Ex9 n = 11		*PDGFRA*/Ex18 n = 4		Wild type n = 6	
Median (range)	59	(18–81)	69	(34–82)	60	(25–64)	35	(17–54)
Sex, *n* (%)								
Male	38	(55.1)	8	(72.7)	2	(50.0)	4	(66.7)
Female	31	(44.9)	3	(27.3)	2	(50.0)	2	(33.3)
Location of primary lesion, *n* (%)								
Esophagus	0	(0)	0	(0)	0	(0)	1	(16.7)
Stomach	26	(37.7)	0	(0)	4	(100)	3	(50.0)
Small intestine	36	(52.2)	10	(90.9)	0	(0)	2	(33.3)
Rectum	6	(8.7)	1	(9.1)	0	(0)	0	(0)
Others	1	(1.5)	0	(0)	0	(0)	0	(0)
Format of metastasis, *n* (%)								
Heterochronous metastasis	54	(78.3)	8	(72.7)	2	(50.0)	5	(83.3)
Concurrent metastasis	15	(21.7)	3	(27.3)	2	(50.0)	1	(16.7)
Location of metastatic lesion, *n* (%)								
Liver	34	(49.3)	5	(45.5)	0	(0)	2	(33.3)
Peritoneum	25	(36.2)	4	(36.3)	4	(100)	1	(16.7)
Liver and peritoneum	10	(14.5)	2	(18.2)	0	(0)	1	(16.7)
Others	0	(0)	0	(0)	0	(0)	2	(33.3)
Number of TKIs								
1	23	(33.3)	3	(27.3)	1	(25.0)	1	(16.7)
2	16	(23.2)	4	(36.4)	0	(0)	1	(16.7)
3 or high	30	(43.5)	4	(36.4)	3	(75.0)	4	(66.7)
Genetic testing for IM-resistant lesions, *n* (%)								
Yes	48	(69.6)	8	(72.7)	3	(60.0)	5	(83.3)
No	21	(30.4)	3	(27.3)	2	(40.0)	1	(16.7)

*Age at diagnosis of unresectable or first recurrence

TKIs: tyrosine kinase inhibitors, IM: imatinib

### Prognostic information derived from secondary gene mutation

Of the 90 patients, 64 were diagnosed with imatinib resistance and underwent GIST-related genetic testing using tissue samples collected before starting secondary treatment (Table 3). Among the 64 patients, the primary mutation was most commonly found in exon 11 of *KIT* (*KIT*/Ex11 group; n = 48, 75.0%). In the *KIT*/Ex11 group, 38 patients (79.2%) had secondary gene mutations in *KIT* exon 13/14 (n = 23, 47.9%) and *KIT* exon 17/18 (n = 15, 31.3%). Ten patients (20.8%) had no secondary mutations. None of the patients in the *KIT*/Ex9, *PDGFRA*/Ex18, or Wild type groups had secondary mutations in GIST-related genes.

**Table 3:** Comparison of secondary gene mutation

Site of secondary mutation, *n* (%)	*KIT*/Ex11 n = 48		*KIT*/Ex9 n = 8		*PDGFRA*/Ex18 n = 3		Wild type n = 5	
*KIT* exon 13/14	23	(47.9)	0	(0)	0	(0)	0	(0)
*KIT* exon 17/18	15	(31.3)	0	(0)	0	(0)	0	(0)
None	10	(20.8)	8	(100)	3	(100)	5	(100)
Genetic testing method^*^, n (%)								
RT-PCR	25	(52.1)	4	(50.0)	2	(66.7)	0	(0)
Direct sanger sequencing	11	(22.9)	3	(37.5)	0	(0)	0	(0)
Gene panel testing	12	(25.0)	1	(12.5)	1	(33.3)	5	(100)

*The method used at the time of imatinib resistance

RT-PCR: reverse transcription-polymerase chain reaction

**Fig. 4:**
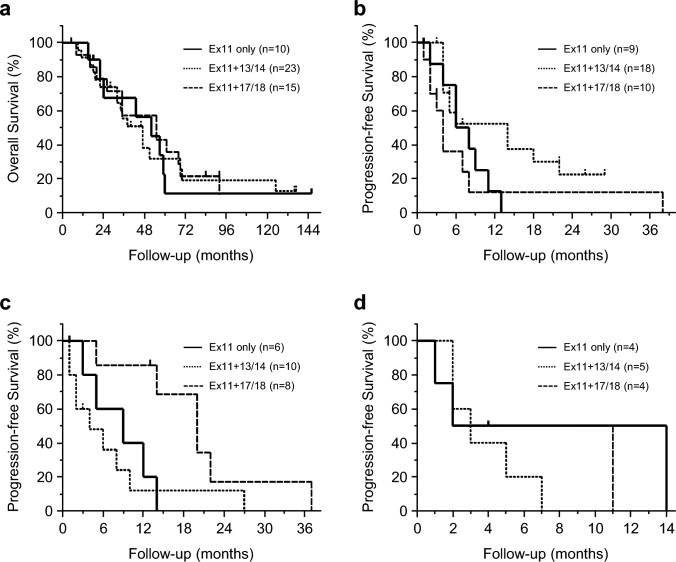
Kaplan–Meier curves for survival by secondary gene mutation. **a** Overall survival after the date of the first documented imatinib resistance. Progression-free survival for **b** sunitinib, **c** regorafenib, and **d** pimitespib. The survival rates were compared using the log-rank test

The prognosis of 48 patients in the *KIT*/Ex11 group was examined according to secondary mutations. In the exon 11 only, exon 11 + 13/14, and exon 11 + 17/18 groups, the median OS after the date of the first documented imatinib resistance was 52, 47, and 55 months, respectively, and there was no significant difference between the three groups (Fig. 4a). In each of the three groups, the median PFS for sunitinib was 7, 14, and 4 months, respectively (Fig. 4b). The exon 11 + 13/14 group tended to have a longer PFS with sunitinib than the exon 11 only group and the exon 11 + 17/18 group (p = 0.074 and 0.056, respectively). In each of the three groups, the median PFS for regorafenib was 9, 4, and 20 months, respectively (Fig. 4c). The exon 11 + 17/18 group showed significantly better results compared to the exon 11 only group and the exon 11 + 13/14 group (p = 0.010 and 0.032, respectively). In each of the three groups, the median PFS for pimitespib was 8, 3, and 6.5 months, respectively (Fig. 4d).

## Discussion

We identified the types and frequencies of mutations in GIST-related genes in patients with unresectable or recurrent GISTs before imatinib treatment and after developing imatinib resistance. Previous reports have shown that the primary mutation of GIST-related genes in primary tumors is a *KIT* mutation in 75–85% of all patients with GISTs, *PDGFRA* mutation in approximately 10%, and wild-type in the remaining 10%, wherein no mutation is observed in either genes [18–20]. In this study, *KIT* mutations were identified for approximately 90% of the gene mutations in patients with unresectable or recurrent GISTs, which aligns with the report by Heinrich et al. [21]. Among the patients in whom genetic testing was performed on imatinib-resistant resected lesions, secondary mutations were not observed in patients with *KIT* exon 9, *PDGFRA* mutations, or the wild-type. In patients with *KIT* exon 11 mutations, secondary mutations were observed in approximately 80% of the patients, which was inferred to affect drug resistance. Nishida et al. reported that secondary mutations in *KIT* occur in approximately 70% of imatinib-resistant patients [22], which is congruent with the current study. Owing to advancements in technology, genetic testing is becoming more convenient in clinical practice; however, the impact of secondary mutations after imatinib resistance on subsequent treatment response and prognosis remains controversial. This study has comprehensively evaluated the association between secondary *KIT* mutations and the efficacy of each therapeutic agent—from second-line sunitinib to fourth-line pimitespib—within a single cohort and clarified its clinical significance.

The median OS after the start of first-line imatinib treatment was 93 months in all patients with unresectable or recurrent GISTs, which was considerably longer than that reported in previous studies [23, 24]. However, the PFS for each TKI was not significantly different from previous data [5–8]. Factors that have contributed to the extension of OS include the establishment of treatment management for unresectable or recurrent GISTs, such as the management of adverse drug events, re-challenge administration of TKIs that have already been used, and resection of drug-resistant lesions.

When prognosis was examined by primary mutation of GIST-related genes, PFS was significantly poorer for exon 9 mutation than for exon 11 mutation in *KIT* in response to imatinib. This is because the *KIT* exon 9 mutation made it difficult for imatinib to bind, making it less effective [21, 25]. In contrast, the PFS of sunitinib-treated patients with the *KIT* exon 9 mutation was significantly better than that of patients with the exon 11 mutation, and many patients responded to sunitinib for a long time. Previous reports have also shown that sunitinib is more effective against GISTs with exon 9 mutations in *KIT*, which often show primary resistance to imatinib [26]. Therefore, if a patient with GIST has an exon 9 mutation in *KIT*, this can be used as a basis to switch to sunitinib as soon as possible when signs of imatinib resistance or intolerance are confirmed. Regarding *PDGFRA* mutations, it has been reported that the D842V mutation in exon 18 has a poor response to imatinib [18, 27], which may be because of structural differences in the binding region preventing imatinib from attaching, leading to resistance [28]. All patients with *PDGFRA* mutations in this study had the D842V mutation in exon 18, and none responded to imatinib, sunitinib, or regorafenib. Furthermore, these drugs target *KIT* and *PDGFRA*, and are not effective in patients with wild-type GISTs [29]. The wild-type patients in this study also had a poor response with all three drugs. Understanding the primary genetic mutations in unresectable or recurrent GISTs may be useful for determining when to change drugs after administering imatinib, and for selecting subsequent treatment methods.

Secondary genetic mutations in the kinase domain have been reported as the cause of imatinib resistance in 40–80% of cases, *KIT* gene amplification in approximately 10% of cases, and alternative activation pathways other than *KIT* and *PDGFRA* in 10–20% of cases [1, 30]. Imatinib competes with adenosine triphosphate (ATP) for the ATP-binding domain of the kinase region of *KIT* and *PDGFRA*, thereby inhibiting the activity of the target molecule. However, if a second genetic mutation occurs in the ATP-binding domain, it becomes resistant to imatinib [31]. In this study, we found secondary gene mutations in the kinase region in approximately 80% of the patients with *KIT* exon 11 mutations. Several previous reports on secondary mutations in GIST-related genes have involved experiments using cell lines and have demonstrated the drug sensitivity of GIST cells with *KIT* secondary mutations [13, 32]. The kinase domain of *KIT* is divided into an ATP-binding domain (exon 13 or 14) and an activation loop (exon 17 or 18). Previous studies have suggested that sunitinib is effective if the gene mutation occurs in the ATP-binding domain, but not in the activation loop [14, 33]. The results of our study did not contradict those of previous studies, and patients with secondary gene mutations in exon 13 or 14 tended to have a longer PFS with sunitinib than those with mutations in exon 17 or 18, as well as those without secondary mutations. Exploratory analyses of the GRID and VOYAGER trials have reported that regorafenib was effective regardless of the presence or absence of secondary *KIT* mutations [15, 34]. In contrast, previous in vitro studies have shown that regorafenib is ineffective in patients with secondary gene mutations in exon 13 [32]. In our study, patients with secondary gene mutations in exon 17 or 18 tended to have a significantly longer PFS with regorafenib than those with mutations in exon 13 or 14, as well as those without secondary mutations. These results suggest that secondary gene mutations may help predict the efficacy of sunitinib and regorafenib.

Furthermore, we examined the efficacy of pimitespib from the perspective of genetic mutations. Pimitespib was recently approved for the treatment of advanced GISTs following the results of the phase III trial CHAPTER-GIST-301 [10], wherein the median PFS for pimitespib was 2.8 months, but in this cohort, it was longer at 4 months. Pimitespib is a highly selective HSP90 inhibitor [35, 36], that reduces HSP90 client proteins, inhibits signal transduction and cell cycle progression, and exerts antitumor effects [37, 38]. When pimitespib was first developed, it was considered effective against GISTs with primary *KIT* and *PDGFRA* mutations. In contrast, Saito et al. showed that in vitro, pimitespib was effective not only in GISTs with primary *KIT* mutations that were sensitive to imatinib, but also in GISTs with secondary *KIT* mutations that were resistant to imatinib [39]. In clinical practice, Kurokawa et al. reported that pimitespib may be effective regardless of the *KIT* mutation status [10], which is congruent with the current study’s findings. Furthermore, a few patients with SDH-deficient wild-type or secondary *PDGFRA* mutations showed a long-term response to pimitespib treatment. The mechanism of action of pimitespib is different from that of TKIs, and it is expected to exhibit a broad antitumor spectrum by reducing multiple client proteins [40, 41]. The efficacy of pimitespib in patients with wild-type or secondary *PDGFRA* mutations remains controversial; however, this study suggests that there may be new therapeutic targets for pimitespib other than those previously reported. The use of pimitespib in earlier lines of treatment for patients who are expected to have a poor response to TKI should be considered, and it may also be necessary to search for new biomarkers that could be used to predict treatment efficacy other than genetic mutations.

This study had some limitations. First, this was a single-center retrospective study. Because of the long period covered in this study, there may have been minimal differences in treatment outcomes because of the historical background. Second, this study showed the results of genetic testing using tissue samples from the initial imatinib-resistant resection as secondary gene mutations. Tissue-based analysis offers superior accuracy in mutation identification and allows for pathological validation of tumor characteristics, however, if there are multiple resistant lesions or if the resistant lesions appeared heterochronously, the secondary gene mutation may be different for each resistant lesion and for each timing. Therefore, although it is practically difficult, genetic testing should be performed each time a drug change is considered. Genetic testing of blood samples using liquid biopsy, which has been gradually performed in recent years, may provide a more comprehensive picture of genetic mutations, even in multiple lesions [42]. At present, the accuracy of liquid biopsy in searching for GIST-related genetic mutations is low, and frequent testing is difficult because of financial restraints [43]. Therefore, if genetic testing using blood samples becomes more accurate and less costly in the future, it may be possible to detect genetic mutations at the time of drug resistance in real-time and provide optimized treatment for each patient.

In conclusion, we clarified the types and frequencies of primary and secondary mutations in GIST-related genes and their clinical relevance in unresectable or recurrent GISTs. Cases of *KIT* exon 9 mutations were not associated with secondary mutations and showed longer PFS with sunitinib than cases of exon 11 mutations. Secondary mutations in exon 13/14 were associated with a trend toward sunitinib sensitivity, while those in exon 17/18 showed greater sensitivity to regorafenib. Genetic profiling throughout the treatment course may help optimize drug selection and timing, paving the way for more personalized therapeutic strategies and improved patient outcomes.

## Supplementary Information

Below is the link to the electronic supplementary material.

Supplementary material 1 (DOCX 26.8 kb)

Supplementary material 2 (PDF 290.0 kb)

## Data Availability

The datasets generated/analyzed during the current study are not publicly available because they contain private information pertaining to the research participants, but are available on request from the corresponding author.
