# Supplementary material for: Clinical impact of primary and secondary KIT mutations on the efficacy of molecular-targeted therapies in gastrointestinal stromal tumors
Source: Gastric Cancer. 2025 Jul 23;28(5):899–910. doi: 10.1007/s10120-025-01639-1 (PMC12378141; doi:10.1007/s10120-025-01639-1)
Supplement: Supplementary file 1 — Supplementary material 1 (DOCX 26.8 kb) [file 10120_2025_1639_MOESM1_ESM.docx]

**Supplementary figure legend**

**Supplementary Fig. 1** Kaplan–Meier curves for survival by primary gene mutation. Overall survival from the initiation of each drug: (a) sunitinib, (b) regorafenib, and (c) pimitespib. Survival rates were compared using the log-rank test.
