# Supplementary figures and images for: Clinical impact of primary and secondary KIT mutations on the efficacy of molecular-targeted therapies in gastrointestinal stromal tumors
Source: Gastric Cancer. 2025 Jul 23;28(5):899–910. doi: 10.1007/s10120-025-01639-1 (PMC12378141; doi:10.1007/s10120-025-01639-1)

supplementary figure1

**a**

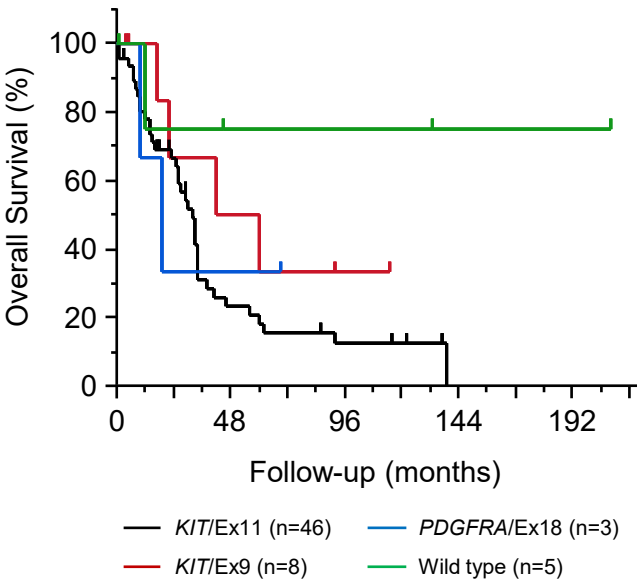

**b**

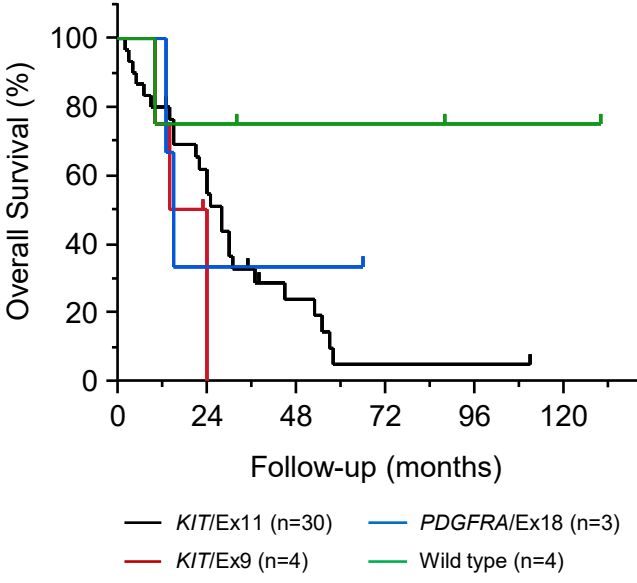

**c**

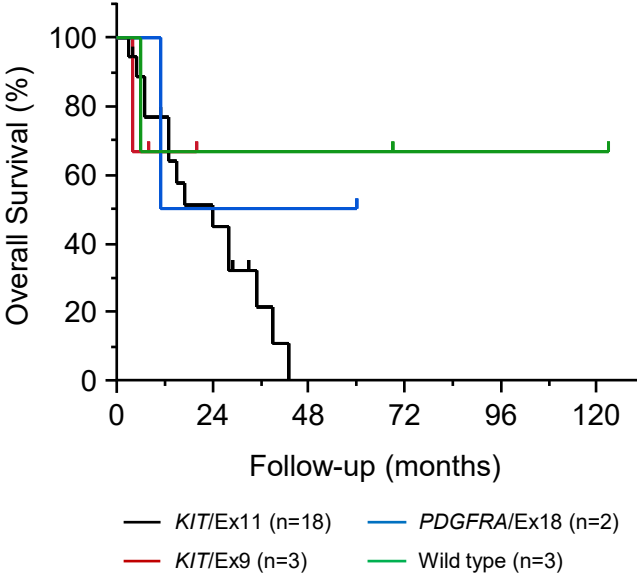

Supplement: Supplementary file 2 — Supplementary material 2 (PDF 290.0 kb) [file 10120_2025_1639_MOESM2_ESM.pdf]
